# DNA methylation of the IGF2/H19 imprinting control region and adiposity distribution in young adults

**DOI:** 10.1186/1868-7083-4-21

**Published:** 2012-11-13

**Authors:** Rae-Chi Huang, John C Galati, Sally Burrows, Lawrence J Beilin, Xin Li, Craig E Pennell, JAM van Eekelen, Trevor A Mori, Leon A Adams, Jeffrey M Craig

**Affiliations:** 1 School of Medicine and Pharmacology, University of Western Australia (UWA), Perth, WA, Australia; 2 School of Paediatrics and Child Health Research, (UWA), Perth, WA, Australia; 3 Telethon Institute for Child Health Research, (UWA), Perth, WA, Australia; 4 Clinical Epidemiology and Biostatistics Unit, Murdoch Children’s Research Institute (MCRI), Royal Children’s Hospital, Melbourne, VIC, Australia; 5 Department of Mathematics and Statistics, La Trobe University, Melbourne, VIC, 3086, Australia; 6 Early Life Epigenetics Group, MCRI and Department of Paediatrics, University of Melbourne, Royal Children’s Hospital, Melbourne, VIC, Australia; 7 Medical Research Foundation Building, Level 4, Rear 50 Murray Street, Perth, WA, 6000, Australia

**Keywords:** Childhood, Fetal programming, DNA methylation, Insulin-like growth factor, Raine Study, Head circumference

## Abstract

**Background:**

The insulin-like growth factor 2 (IGF2) and *H19* imprinted genes control growth and body composition. Adverse *in*-*utero* environments have been associated with obesity-related diseases and linked with altered DNA methylation at the *IGF2*/*H19* locus. Postnatally, methylation at the *IGF2*/*H19* imprinting control region (*ICR*) has been linked with cerebellum weight. We aimed to investigate whether decreased *IGF2*/*H19 ICR* methylation is associated with decreased birth and childhood anthropometry and increased contemporaneous adiposity.

DNA methylation in peripheral blood (n = 315) at 17 years old was measured at 12 cytosine-phosphate-guanine sites (CpGs), analysed as Sequenom MassARRAY EpiTYPER units within the *IGF2*/*H19 ICR*. Birth size, childhood head circumference (HC) at six time-points and anthropometry at age 17 years were measured. DNA methylation was investigated for its association with anthropometry using linear regression.

**Results:**

The principal component of *IGF2*/*H19 ICR* DNA methylation (representing mean methylation across all CpG units) positively correlated with skin fold thickness (at four CpG units) (*P*-values between 0.04 to 0.001) and subcutaneous adiposity (*P* = 0.023) at age 17, but not with weight, height, BMI, waist circumference or visceral adiposity. *IGF2*/*H19* methylation did not associate with birth weight, length or HC, but CpG unit 13 to 14 methylation was negatively associated with HC between 1 and 10 years. *β*-coefficients of four out of five remaining CpG units also estimated lower methylation with increasing childhood HC.

**Conclusions:**

As greater *IGF2*/*H19* methylation was associated with greater subcutaneous fat measures, but not overall, visceral or central adiposity, we hypothesize that obesogenic pressures in youth result in excess fat being preferentially stored in peripheral fat depots via the *IGF2/H19* domain. Secondly, as *IGF2*/*H19* methylation was not associated with birth size but negatively with early childhood HC, we hypothesize that the HC may be a more sensitive marker of early life programming of the IGF axis and of fetal physiology than birth size. To verify this, investigations of the dynamics of *IGF2*/*H19* methylation and expression from birth to adolescence are required.

## Background

Insulin-like growth factor 2 (IGF2) is one of two ligands within the IGF axis central to the control of somatic growth, especially in early life, through a balance of cell proliferation and apoptosis. IGF2’s major signal transduction is via activation of the IGF1 receptor, which mediates anabolic effects in adults. Activation of this receptor has systemic growth promoting effects, particularly on skeletal muscle, bone and neural tissue [1]. A critical timing of action of IGF2 appears to be antenatal, with *Igf2*-deficient mice being severely growth restricted at delivery [2,3]. IGF2 also acts as part of the IGF signaling pathway to regulate the postnatal growth of somatic tissues, including the brain [4] and, later in life, changes in the IGF axis manifest in altered fat and body composition. Decreased expression of *Igf2* is associated with increased fat mass, despite hypophagia, in a mouse model [5]. In adult humans, lower circulating IGF2 levels have been associated with increased risk of weight gain and obesity [6]. Furthermore, associations have been found between *IGF2* genotype and obesity in humans in some studies [7-12] or with height but not obesity [13].

The *IGF2*/*H19* locus is involved in fetal programming through DNA methylation in rodents [14] and humans [15]. It is perhaps the best studied of all identified imprinted genetic loci [16]. In imprinting, genes are expressed in a parent-of-origin manner, under the control of imprinting control regions (ICRs), which are themselves differentially methylated between maternal and paternal alleles [17]. In humans, the *IGF2* gene and adjacent *H19* non-coding RNA are clustered in an imprinted region on the short arm of chromosome 11. The cluster is regulated by an ICR, which contains multiple binding sites for the insulator protein CTCF, the critical site referred to as CTCF6 [18]. An unmethylated *IGF2*/*H19* ICR on the maternal allele allows binding of CTCF which blocks transcription of *IGF2* and activates *H19*, in turn inhibiting growth. Conversely, on the paternal allele, *IGF2*/*H19* ICR methylation activates *IGF2* transcription and silences *H19*. In general, *H19* has a growth restraining effect and *IGF2,* a growth promoting effect. Accurate DNA methylation of the *IGF2*/*H19* locus is important for growth [19] and disorders of imprinting mediated by DNA methylation in this region (Russell Silver and Beckwith Wiedemann syndromes) are associated with clear phenotypes of altered growth [20,21]. Russell Silver Syndrome is usually associated with hypomethylation of the ICR, which may lead to a tissue specific loss of *IGF2* gene activity and increased activity of the *H19* gene [22]. The overgrowth Beckwith-Wiedemann syndrome is associated with hypermethylation of the ICR, leading to increase of *IGF2* and decrease in *H19* activity.

Adverse intrauterine events have been associated with subsequent obesity and metabolic disease in so-called “fetal programming” [23], but the underlying mechanisms for this are not well understood. One major candidate is epigenetic dysregulation, of which DNA methylation is the most studied mechanism, and which has been shown to have a role in animal models of fetal programming [24,25]. For example, a recent study showed that a maternal low protein diet during gestation in rats resulted in greater DNA methylation of the *IGF2*/*H19 ICR* in liver tissue, which interestingly was reversed with the addition of folate to the maternal diet [14].

The first empirical evidence in a human population confirming that altered DNA methylation is associated with fetal programming was shown elsewhere within the *IGF2*/*H19* locus. Offspring exposed to maternal starvation early in gestation during the Dutch Famine in the 1940s had decreased methylation at the *IGF2 DMR,* an imprinted region of differential methylation within the *IGF2* gene [15] which was accompanied by obesity later in life [26,27]. In addition, periconceptional folic acid supplementation has also been associated with increased DNA methylation levels at the *IGF2* DMR (upstream of exon 3) [28]. Such supplementation has also been associated with decreased methylation at the ICR region CTCF6 [29] that has been linked to overweight status at age one year [30]. We hypothesize that this overweight status may persist to age 17 years.

In the present study, our aim was to investigate whether at 17 years of age increased methylation of the *ICR* of the *IGF2*/*H19* locus is associated with greater adiposity (BMI, abdominal and visceral adiposity) and decreased subcutaneous fat, and whether decreased earlier measures of anthropometry from birth onwards are associated with increased *IGF2*/*H19 ICR* methylation.

## Results

### General characteristics

**Figure 1:**
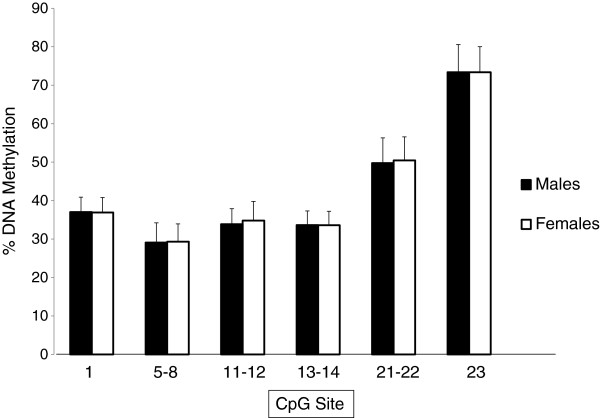
**Percentage DNA methylation of each CpG unit of the *ICR of the IGF2*/*H19 *locus.** Males are shown in shaded and females in open bars. Error bars show standard deviation.

General characteristics of the 17-year-old participants used in the current study are shown in Table 1. Of the 315 participants derived from this population cohort, none were diagnosed with pre-existing type 1 or 2 diabetes, the metabolic syndrome [31] or Beckwith-Wiedemann Syndrome. A comparison with all 17-year-old Raine study participants is shown in Additional file 1: Table S1. Those with DNA methylation measured showed minor differences, being younger by 2.5 months and having on average a greater BMI of 0.8 kg/m^2^. There was no difference with respect to gestational age at delivery, gender or birth weight. Figure 1 shows the amount of DNA methylation for each of the 6 *IGF2*/*H19 ICR* CpG units investigated separated by sex. DNA methylation ranged between 30 to 70%, and there was no significant difference in the amount of DNA methylation at this locus in males and females (all *P* >0.05).

**Table 1:** General characteristics of the individuals at age 17 years for which DNA methylation was measured

	**Male**		**Female**	
**n**	**162**		**144**	
	**Mean**	**(SD)**	**Mean**	**(SD)**
Age (years)	16.4	(0.5)	16.4	(0.5)
Weight (kg)	59.4	(14.8)	58.6	(14.7)
Height (cm)	166.7	(8.4)	162.6	(6.2)
BMI (kg/m^2^)	21.2	(4.5)	22.1	(5.1)
Waist circumference (cm)	77.6	(12.6)	75.7	(12.0)
Gestational age (weeks)	39.0	(1.7)	39.0	(1.6)
	%		%	
Family income (%)				
≤$25,000	4.6		8.6	
$25,000 to $50,000	13.8		24.2	
$50,001 to $104,000	41.5		43.8	
≥$104,001	36.2		23.4	
Ethnicity (% whose mothers are Caucasian)	91		88	

### *IGF2*/*H19 ICR* DNA methylation and birth parameters

We measured DNA methylation at the *IGF2*/*H19 ICR* in peripheral blood from the 315 17-year-old participants using the Sequenom MassARRAY EpiTYPER platform, which is based on bisulphite conversion of only non-methylated cytosines to uracil, base-specific cleavage (in this case, at the thymine base) and mass spectrometry. This produced quantitative methylation data for 12 CpGs clustered into 6 CpG units (Figure 1), corresponding to quantifiable fragments each containing 1 to 4 CpGs. No association was seen between *IGF2*/*H19 ICR* DNA methylation at age 17 years and birth weight, birth length or birth head circumference (all *P* >0.05). (Additional file 1: Tables S2 and S3).

### Association between *IGF2*/*H19 ICR* DNA methylation and anthropometry at age 17

All further results are presented in units of sex-specific z-scores to simplify models such that gender need not be included in the models, since anthropometry differed by sex. To allow conversion of measures of association into original units, Table 2 gives the sex-specific standard deviations for each of the measurements at 17 years (a one unit change in z-score for a given variable corresponds to a one standard deviation change in units of the underlying variable). Results from univariate regression analysis of the association between *IGF2*/*H19 ICR* methylation at each CpG unit and anthropometric measurements are shown in Additional file 1: Table S2.

**Table 2:** One standard deviation for each anthropometry and DNA methylation variable

**1 Standard deviation**	**Males**	**Females**
Weight (kg)	14.6	13.3
Height (cm)	7.3	6.5
BMI (kg/m^2^)	4.2	4.6
Waist (cm)	10.9	11.4
**Skin fold thickness (mm)**		
Subscapular	6.5	6.7
Suprailiac	8.6	7.8
Abdominal	9.7	7.9
Triceps	6.2	6.2
**Fat thickness (cm)**		
Subcutaneous	10.5	11.1
Visceral	10.7	8.9
Birth weight (g)	621	613
Birth length (cm)	2.9	3.0
Birth head circumference (cm)	2.0	2.0
Head circumference at age 1 (cm)	1.3	1.3
Head circumference at age 2 (cm)	1.4	1.4
Head circumference at age 3 (cm)	1.4	1.4
Head circumference at age 5 (cm)	1.5	1.4
Head circumference at age 8 (cm)	1.5	1.5
Head circumference at age 10 years (cm)	1.5	1.7
Principal components		
Principal component 1 (score)	1.8	1.6
Principal component 2 (score)	1.3	1.1
CpG 1 (%)	3.9	3.9
CpG 5 to 8 (%)	5.1	4.6
CpG 11 to 12 (%)	4.0	5.0
CpG 13 to 14 (%)	3.7	3.6
CpG 21 to 22 (%)	6.6	6.1
CpG 23 (%)	7.2	6.7
Mean H19 (%)	4.6	6.5
Average of all SD	5.1	5.0

A change of one in a z-score for a given variable corresponds to a one standard deviation change in units for that variable.

**Figure 2:**
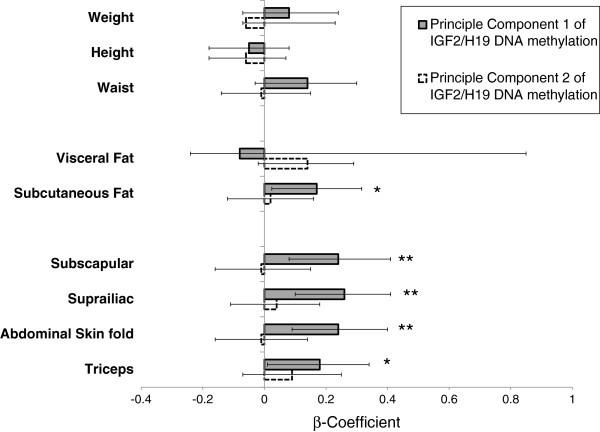
**Linear regression showing the effect of IGF2/H19 methylation principal components upon age 17 years anthropometry.** The β-coefficient and 95% confidence interval of the principle components of IGF2/H19 methylation are shown for a unit change in anthropometric measure. Principal component 1 (indicated by gray shaded bars and solid confidence interval lines) reflects within subject mean of IGF2/H19 methylation. Principal component 2 (indicated by open bars and dotted confidence interval lines) reflects within-subject standard deviation of the IGF2/H19 methylation. * indicates a *P*-value <0.05 and ** indicates a *P*-value <0.005.

Figure 2 shows the association between principal components 1 (representing the mean of methylation across the CpG units) and 2 (representing the within-subject standard deviation) of *IGF2*/*H19* methylation with anthropometric measurements at age 17 years. No relationship was seen between DNA methylation and weight, height and BMI or waist circumference. A positive association was seen between principal component 1 of DNA methylation and three measures of skin-fold thicknesses (subscapular, suprailiac, triceps and abdominal) at age 17. A one z-score increase in *IGF2*/*H19 ICR* DNA methylation principal component 1 was associated with a 0.24 (95% CI 0.09 to 0.40) standard deviations increase in abdominal skin-fold thickness. This is equivalent to a change in 2.5 mm in boys and 2.0 mm in girls. Since principal component 1 reflects average methylation across the six CpG units, a one z-score increase in principal component 1 corresponds to an approximate 5.1% (males) and 5.0% (females) increase in average DNA methylation in this locus. A positive association was also seen between *IGF2*/*H19 ICR* DNA methylation principal component 1 and subcutaneous fat thickness, but not with visceral fat thickness (Figure 2).

### Association of *IGF2*/*H19* ICR methylation with serial head circumference between 1 and 10 years

Head circumference was measured at six time points between 1 and 10 yearsof age, necessitating a variance component adjustment using STATA’s vce option, to account for the correlation between repeated measures within a subject. On univariate regression, methylation at two CpG units was associated with aggregated head circumference (Table 3). One SD increase in *IGF2*/*H19* CpG 13 to 14 was associated with a decrease in 0.12 z-score of head circumference. This equates to a 3.4% increase in methylation at this CpG unit being associated with a decrease in head circumference of 18 mm (*P* = 0.006). Although four out of five of the remaining CpG units also had negative coefficients, albeit not reaching statistical significance (*P* = 0.089 to 0.27), neither principal component 1 nor 2 was associated with aggregated head circumference.

**Table 3:** Univariate regression of head circumference on IGF/H19 ICR

	**Coefficient**	***P*-value**	**95% CI**
CpG1	−0.08	0.12	−0.17 to 0.02
CpG5-8	−0.05	0.27	−0.14 to 0.04
CpG11-12	−0.9	0.089	−0.19 to 0.01
CpG13-14	−0.12	0.006	−0.21 to −0.04
CpG21-22	−0.07	0.11	−0.16 to 0.02
CpG23	0.04	0.38	−0.05 to 0.13
Principal component 1	−0.07	0.14	−0.16 to 0.02
Principal component 2	0.07	0.17	−0.03 to 0.18

Head circumference is associated with each of the six CpG units and on principal components 1 and 2 of DNA methylation. Within subject correlation across time between 1 and 10 years of age was accounted for using a sandwich estimator (vce option in STATA).

## Discussion

This study showed no evidence that *IGF2*/*H19 ICR* DNA methylation in peripheral blood at age 17 years is associated with birth anthropometry. On the other hand, a negative association with aggregated head circumference from 1 to 10 years and a positive association with skin fold thicknesses from multiple sites and subcutaneous adiposity at 17 years were observed.

Our study found repeated positive associations of greater *IGF2*/*H19 ICR* methylation with greater subcutaneous adiposity measured by multiple methods, including direct ultrasound and by calliper assessed skin folds at multiple body sites. The positive association of *IGF2*/*H19 ICR* methylation with subcutaneous adiposity in this study adds to the recent observations by Perkins *et al.* showing greater *IGF*/*H19 ICR* methylation in overweight or obese one-year-olds compared to normal weight counterparts [30]. The current study shows that an association between adiposity and methylation at this site persists to young adult life. It is also consistent with current understanding that the IGF axis increases the proportion of subcutaneous fat to visceral fat [32,33], particularly in younger age groups. In neonates, levels of *IGF1* protein have been positively associated with subcutaneous fat and sum of skin folds, but not with visceral fat [32]. Interestingly, Russell Silver syndrome patients, who have decreased expression of *IGF2*, have a striking lack of subcutaneous fat as part of their generalized growth retardation [34]. Furthermore, genome wide association studies have identified the 11p15.5 region inclusive of the *IGF2* gene to be associated with abdominal subcutaneous fat [35,36].

In general, activation of the IGF axis decreases overall fatness. Many [7,9-12], but not all [13] candidate studies have shown that *IGF2* polymorphisms are associated with overall fatness. Specific to visceral adiposity, some [37], but not all [38] studies show an inverse correlation with IGF1 protein levels. Despite this, contrary to our initial hypotheses, no relationships were seen between *IGF2*/*H19 ICR* methylation and either BMI, waist circumference or visceral fat at age 17 years. BMI is relatively easy to measure, but does not always correlate with total fat mass and may be confounded by muscle mass [39]. As activation of the IGF axis also increases muscle mass [40], the lack of discrimination of the BMI variable for fat and muscle body compartments may account for its poor correlation with *IGF2 ICR* methylation.

The lack of associations between *IGF2*/*H19* ICR methylation and visceral fat or abdominal circumference (a marker for central adiposity) are also interesting to note. We speculate that the lack of association seen might be due to the relative youth of our cohort where the early response to obesogenic influences results in preferential fat deposition in the protective subcutaneous compartment [41,42], imparting a lesser metabolic risk than the visceral compartment [43]. At 17 years of age, DNA methylation at the *IGF2*/*H19 ICR* does not appear to be associated with detrimental fat distribution. However, no definitive conclusion can be drawn as to whether the altered DNA methylation is a consequence or cause of the altered subcutaneous adiposity in our study.

Based on the knowledge that suboptimal intrauterine environments leading to fetal programming are associated with epigenetic changes [14,15], we hypothesized that birth anthropometry would be associated with *IGF2*/*H19* ICR methylation. Contrary to this, we did not detect an association of DNA methylation with any birth size parameters. This finding is not so unexpected given that fetal programming caused by altered intrauterine environment has been shown repeatedly to occur without disturbance in birth size [15,44,45]. Sheep experiments isolating the effect of maternal under- and over-nutrition in the periconceptional period, show that weight at birth is not affected [45], despite subsequent development of increased obesity and adverse metabolic parameters. In the Dutch Famine cohort, only modest differences in birth weight of 50 to 100 g were observed for exposed individuals overall [46] and epigenetic differences were only present in those of normal birth weight after being exposed to periconceptional starvation [15]. Those with exposure to starvation later in gestation did demonstrate reduced birth weight, but in the absence of changes in DNA methylation [15]. On a molecular level, epigenetic marks may be particularly vulnerable during very early development [47] a finding supported by experimental evidence of altered *H19* methylation and expression in mouse 2 cell embryos cultured to the very early stage of blastocysts [48]. In addition, there is evidence that intrauterine growth retardation may not be associated with epigenetic changes at *IGF2*[49-51]. This suggests that birth weight may be a poor surrogate for a suboptimal environment during pregnancy when testing for fetal programming via DNA methylation mechanisms. It is likely that the intrauterine effects that cause DNA methylation changes in a modern, well-nourished population are subtle and do not cause great birth weight changes.

In our study, head circumference (HC) at birth was not correlated with *IGF*/*H19 ICR* DNA methylation at 17 years. However, between 1 and 10 years HC was negatively correlated with *IGF*/*H19* ICR DNA methylation (in all CpG units except for CpG 23). The key role of the IGF axis and methylation of its related genes in head size maintenance is evident; for example, *IGF1*-deficient subjects treated with exogenous IGF1 show a striking increase in head circumference [52]. Adult brain size has been shown to relate positively to *IGF2* DMR2 (exon 9) methylation and be negatively related to *IGF*/*H19 ICR* (at the CTCF3 region 2kb from our ICR assay) DNA methylation in DNA extracted from the cerebellum [53,54]. Although the region within the ICR assayed in the latter study did not overlap with our assay, the negative direction of association was the same. Further, dietary interventions aimed at modifying DNA methylation (periconceptional folate supplementation) have been associated with both increases in *IGF2 DMR* DNA methylation [28] and head size [55]. *In-utero* head circumference is genetically [56] and, possibly also, epigenetically controlled.

The reason that an association was detectable in infancy/childhood, but not immediately post-delivery may be due to compromise in the accuracy of HC measurements by molding that inevitably occurs with a non-breech delivery. In addition, maternal uterine constraint may play a role, whereby both gain in size and stunting only become apparent after a period of time outside the limiting influences of the womb [57]. Differences might also be due to variation in the mechanisms of *IGF2*/*H19* imprinting between blood and brain [58]. Alternatively, birth size may truly have no linear relationship with *IGF2*/*H19* methylation. This is plausible given that fetal programming can occur without change in birth size [45]. It is also possible that in our cohort, fetal programming may have influenced DNA methylation at the *IGF2*/*H19* locus, specifically *in utero*, for which there is prior evidence in relation to certain early life environments [28,29,59].

No conclusion can be drawn as to whether the altered DNA methylation is a consequence or cause of the altered head size in our study. The DNA methylation was measured at a later age than some of the longitudinal anthropometry measurements. Nevertheless, there is evidence that imprinted domains, such as the studied *IGF2*/*H19 ICR* (having been established either in germ cells in the previous generation or somatic tissues *in utero*), are relatively stable over time postnatally [60], into late adulthood [61,62] and equivalent across tissues in humans [60]. As further indirect confirmation we have shown that Ollikainen *et al*.’s [18] measurements of the same region at birth are comparable to measurements at age 14 years in this study.

This is, to our knowledge, the first study to investigate the association between DNA methylation and longitudinal growth. Although RNA was not available from study members, we suggest that, due to the previously demonstrated association between *IGF2*/*H19* DMR methylation, expression and levels of IGF2 protein [63], this association will extend to levels of IGF2 transcripts and protein. A strength of the study is the large number of subjects and the serial prospective anthropometry. However, a limitation of the study is that DNA methylation has only been measured at one time point at age 17. Although methylation levels in peripheral blood (as used in our study) appear very similar to those seen at birth in the same tissue [18] and *IGF2*/*H19* imprinting is likely to be stable over time in adults [60,62], a greater understanding is required regarding the dynamics and influences on postnatal methylation in childhood and adolescence.

Further, the importance of tissue in which the DNA methylation is measured needs to be considered, as we have found that effects of intrauterine exposures can be different and even opposite in different tissues (JMC, unpublished data). DNA has been derived from peripheral blood samples in this study for ethical and practical reasons. To our knowledge, no study has compared methylation in blood, adipose and brain tissues, although altered DNA methylation has been observed with obesity in peripheral blood [64]. Furthermore, the region we studied (*H19 CTCF6 DMR)* has shown minimal variation in methylation between tissues, while *IGF2 DMR* showed greater variation [18].

Other limitations are the subset of the Raine cohort used for this study, which is biased to higher BMI and extremes of cortisol measurements. Although adjustment for serum cortisol and adrenocorticotropic hormone (ACTH) in the models did not alter the direction or significance of our findings (data now shown), the significance of our findings would be enhanced with further analysis of the entire cohort and in other cohorts.

In a similar manner to genetic linkage, interaction between CpG sites needs to be resolved to clarify interpretation of epigenetic studies [65,66]. The statistical challenge of modeling potentially highly-correlated CpG sites raises the issue of collinearity, whereby use of multiple regression to isolate the individual contribution of each CpG site to the association under study becomes difficult. To overcome this issue, we took two approaches. First, we performed univariate regression, whereby each of the six CpG units was investigated individually. This allowed identification of individual CpG units potentially playing a greater or lesser role in the phenotypes, but has the drawback of multiple testing and the potential of for false positives. The second complementary approach, principal components analysis, minimized multiple testing, while reflecting average methylation across all the six CpG units. Both methods were applied to investigate the effect of *IGF2* DMR methylation on the outcome of anthropometry at 17 years (Figure 2) and the outcome of head circumference (Table 3). For anthropometry at 17 years, the principal components analysis confirms the univariate results, indicating a general association with methylation in the *IGF2* DMR region studied. For head circumference, the associations with individual CpG units appear to be of differing signs for different CpG units, indicating that any association with methylation in the region studied is more likely to be CpG unit specific.

## Conclusions

In summary, this study shows that *IGF2*/*H19 ICR* DNA methylation is associated with a particular pattern of fat distribution in early adult life and with altered head circumference throughout childhood. Our finding of a greater DNA methylation with greater skin fold thicknesses is corroborated by the similar relationship we observed with subcutaneous fat thickness measured by direct ultrasound. It suggests that *IGF2*/*H19 ICR* DNA methylation may be associated with altered trajectories of body composition. The role of *IGF2*/*H19* DNA methylation on fetal programming on development of obesity-related diseases needs clarification with longitudinal measurements of DNA methylation paired with serial growth measurements.

## Methods

### Ethics statement

Ethics approval was obtained from the relevant institutional Human Ethics Committees (Princess Margaret Hospital). Written informed consent was obtained at recruitment and at each follow-up from the mother or legal guardian as well as from the adolescent at 17 years.

### Population

This study was undertaken on the West Australian Pregnancy (Raine) Study described elsewhere [67]. The Raine study recruited 2,900 women in 1989 to 1990 who delivered 2,868 live births. Follow-up of the offspring has been undertaken at 1, 2, 3, 5, 8, 10, 14 and 17 years. A subset was selected at age 17 years (n = 315) for measurement of *IGF2*/*H19 ICR* DNA methylation. This was a pilot study for which samples were selected with preponderance for individuals with higher BMI and equally across tertiles of salivary cortisol.

### Anthropometry measurements at 17 years

At the 17-year follow-up, weight was measured using the Wedderburn Chair Scales (to the nearest 100 g) and height was measured by the Holtain Stadiometer (to the nearest 0.1 cm). Body mass index (BMI) was calculated by weight (kg)/height (m)^2^. Waist circumference was measured to the nearest cm. Head circumference measurements during childhood were measured at 1, 2, 3, 5, 8 and 10 years of age.

Skin folds were measured with calipers (Holtain Tanner/Whitehouse skin fold calliper, Holtain, Crosswell, UK), taking a generous pinch of skin and subcutaneous tissue with the body part in the relaxed state, taking care not to include underlying muscle. The calipers were left on for three seconds and skin fold thickness recorded to the nearest 0.1 mm. The sub-scapular skin folds were assessed from a vertical immediately beneath the apex of the left scapula with the arm hanging by the child's side. The supra-iliac skin fold was measured from a vertical skin fold immediately above the left anterior superior iliac spine. The abdominal skin fold was measured from a vertical skin fold immediately to the left of the umbilicus. The triceps skin fold was measured from a vertical skin fold on the posterior aspect of the upper arm, halfway between the olecranon and the acromion with the arm hanging by the individual's side.

### Ultrasound measurement of fat thickness

Ultrasound (Siemens Antares, Mountain View, CA, USA) was performed by trained ultrasonographers. Adipose tissue thickness was measured using validated standardized criteria [68]. Visceral adipose tissue thickness was measured as the distance between the anterior wall of the aorta and the internal face of the rectus abdomens muscle perpendicular to the aorta. Subcutaneous fat thickness was measured as the thickness of the fat tissue between the skin-fat interface and the linea alba. Care was taken to avoid subcutaneous fat tissue compression. A single specialist radiologist who was blinded to the clinical and laboratory characteristics of the subjects interpreted the ultrasound images.

### Birth anthropometry

A midwife examined babies 24 to 72 hours after birth. Length was measured with a Harpenden Neonatometer to nearest 0.1 cm. Head circumference was measured to nearest 0.1 cm and birth weight was measured to nearest gram.

### DNA methylation

DNA extraction, bisulphite conversion and locus-specific methylation analysis DNA was prepared from whole blood cells by standard phenol:chloroform extraction and ethanol precipitation as described previously [18]. A total of 500 ng to 1 μg genomic DNA was bisulphite converted using the MethylEasy Exceed Rapid Bisulphite Modification Kit (Human Genetic Signatures, North Ryde, NSW, Australia) and a region of the *IGF2*/*H19 ICR* containing the sixth CTCF binding site (chr11:2,020,978-2,021,293; NCBI Genome Browser assembly 36) amplified as described previously [18]. DNA methylation was measured using the MassARRAY EpiTYPER (SEQUENOM Inc., Herston, QLD, Australia). This method relies on base-specific cleavage and mass spectrometry to measure the level of methylation in DNA fragments (referred to herein as units) containing one or more CpGs [69]. To reduce methylation variability introduced during PCR, at least two replicate amplifications were performed in all instances and data were subjected to stringent cleaning steps as outlined previously [18]. In brief, CpG analytic units that failed to produce data for >30% of samples were discarded, and samples with more than 30% missing data points within an amplicon had all methylation values for that sample set to missing. Finally, technical replicates showing ≥5% absolute difference from the median value of a set of technical replicates were set to missing and only samples with at least two successful technical replicates were analyzed [18].

### Statistical methods

Data were analyzed using SPSS version 19.0, Chicago, IL, USA and STATA version 12 (StataCorp. 2011. Stata Statistical Software: Release 12. College Station, TX, USA: StataCorp LP.) Comparisons of participants at the 17-year follow-up with and without methylation were performed using Chi-square for categorical variables and t-tests for continuous variables, depending on whether the assumption of normality was plausible.

All anthropometric measurements (birth weight and height; head circumference at birth, 1, 2, 3, 5, 8 and 10 years; weight, height, BMI, waist circumference, skinfold and fat thicknesses at 17 years of age) were converted to sex-specific z-scores. Because of the uncertain nature of any relationship between DNA methylation and anthropometry, whether general or involving specific CpG units only, we first tested for a general association in this region. To facilitate this investigation and, at the same time, to reduce the number of regression coefficients being estimated, a principal components analysis was performed on the CpG unit variables to generate component scores that were independent of each other. Two principal components were constructed accounting for 49% and 24% of the variance, respectively, and 73% of the total variance. In our data, the first component represented the within-subject mean of methylation across the CpG units, while the second component represented the within-subject standard deviation (SD) of methylation (correlation between the second component and within-subject SD was 0.98; eigenvectors for these two principal components are given in Additional file 1: Table S4). Principal components were converted to sex-specific z scores and then used in a multivariable linear regression analysis to examine association with anthropometry at 17 years. Head circumference was measured at multiple time points, necessitating a variance component adjustment using STATA’s vce option, to account for the correlation between repeated measures within a subject. In addition to assessment of a general association between DNA methylation and anthropometry, an exploratory univariate linear regression analysis looking at the effect of CpG unit DNA methylation on each anthropometric measurement was undertaken to explore the individual association of each CpG unit with the anthropometric outcome. A subsequent multivariable model to separate the individual contributions of the various CpG units was not viable due to strong correlation between CpG units. Significance criteria was set at alpha = 0.05. Due to the moderate number of potentially positively-correlated tests, it was deemed that adjustment for the increased risk of type I error arising from multiple comparisons in the univariate analyses was not feasible (simple corrections such as the Bonferroni adjustment are known to have low statistical power and *P*-value distribution based methods such as the False Discovery Rate rely on a substantial number independent test, or clusters of tests, being performed [70].

## Abbreviations

ACTH: adrenocorticotropic hormone; BMI: body mass index; CpG: cytosine-phosphate-guanine; CTCF: CCCTC-binding factor; DMR: differentially methylated region; DNA: deoxyribonucleic acid; HC: head circumference; ICR: imprinting control region; IGF2: insulin like growth factor 2; kb: kilobases; PCR: polymerase chain reaction; vce: variance estimator.

## Competing interests

The authors declare that they have no competing interests.

## Authors’ contribution

RCH initiated and conceived the study, undertook and interpreted analyses, and drafted the manuscript. JCG provided analytic guidance, data interpretation and co-drafted the manuscript. SB provided statistical guidance, while LJB provided data interpretation and manuscript review. XL performed epigenetic laboratory studies. CP reviewed this manuscript and supervises the Raine Study. JAM jointly conceived the project and reviewed the manuscript. TAM reviewed the manuscript. LAA provided ultrasound measurements and JMC supervised the epigenetic work and co-drafted the manuscript. All authors read and approved the final manuscript.

## Supplementary Material

Additional file 1 **Table S1. **Comparison of participants at 17-year follow-up who did and did not have DNA methylation measured. **Table S2.** Results of univariate regression of skinfold, anthropometric, fat and birth measures on DNA methylation at age 17. **Table S3.** Multivariate models for birth anthropometry. **Table S4.** Eigenvectors for first two Principal Components of DNA Methylation. Click here for file
