# Supplementary material for: DNA methylation of the IGF2/H19 imprinting control region and adiposity distribution in young adults
Source: Clin Epigenetics. 2012 Nov 13;4(1):21. doi: 10.1186/1868-7083-4-21 (PMC3507742; doi:10.1186/1868-7083-4-21)
Supplement: Additional file 1 — Table S1. Comparison of participants at 17-year follow-up who did and did not have DNA methylation measured. Table S2. Results of univariate regression of skinfold, anthropometric, fat and birth measures on DNA methylation at age 17. Table S3. Multivariate models for birth anthropometry. Table S4. Eigenvectors for first two Principal Components of DNA Methylation. [file 1868-7083-4-21-S1.docx]

Supplementary Table 1: Comparison of participants at 17 year follow up who did and did not have DNA methylation measured.

|  | Participants With DNA methylation assayed |  | Participants Without DNA methylation assayed |  | p value |
| --- | --- | --- | --- | --- | --- |
|  | mean | (SD) | mean | (SD) |  |
| n | 302 |  | 1251 |  |  |
| age | 16.4 | (0.5) | 16.6 | (0.5) | <0.001 |
| sex (male) | 52.6 |  | 50.3 |  | 0.51 |
| Birthweight (kg) | 3.36 | (0.55) | 3.32 | (0.59) | 0.256 |
| Gestational Age (week) | 39.5 | (1.7) | 39.3 | (2.1) | 0.074 |
|  |  |  |  |  |  |
| BMI | 23.5 | (5.4) | 22.8 | (4.1) | 0.011 |
| weight (kg) | 59.0 | (14.7) | 57.4 | (12.8) | 0.044 |
| height (m) | 1.72 | (0.09) | 1.72 | (0.09) | 0.28 |

Supplementary Table 2: Results of univariate regression of skinfold, anthropometric, fat and birth measures on DNA methylation at age 17.

| **Anthropometric measurement** | **CpG** | **β coefficient** | **P Value** | **(95% CI)** |
| --- | --- | --- | --- | --- |
| **Skinfold thickness** |  |  |  |  |
| Subscapular | CpG1 | 0.160 | 0.078 | (-0.02 to 0.34) |
|  | CpG5-8 | 0.10 | 0.23 | (-0.06 to 0.27) |
|  | CpG11-12 | 0.26 | 0.005 | (0.08 to 0.45) |
|  | CpG13-14 | 0.18 | 0.046 | (0.003 to 0.361) |
|  | CpG21-22 | 0.19 | 0.023 | (0.03 to 0.35) |
|  | CpG23 | 0.07 | 0.38 | (-0.09 to 0.23) |
|  |  |  |  |  |
| Suprailiac | CpG1 | 0.17 | 0.043 | (0.005 to 0.340) |
|  | CpG5-8 | 0.05 | 0.52 | (-0.11 to 0.21) |
|  | CpG11-12 | 0.28 | 0.002 | (0.10 to 0.45) |
|  | CpG13-14 | 0.22 | 0.012 | (0.05 to 0.39) |
|  | CpG21-22 | 0.23 | 0.004 | (0.07 to 0.38) |
|  | CpG23 | 0.11 | 0.16 | (-0.04 to 0.26) |
| Abdominal Skin fold | CpG1 | 0.16 | 0.051 | (-0.001 to 0.33) |
|  | CpG5-8 | 0.09 | 0.24 | (-0.06 to 0.25) |
|  | CpG11-12 | 0.26 | 0.003 | (0.08 to 0.43) |
|  | CpG13-14 | 0.23 | 0.006 | (0.07 to 0.40) |
|  | CpG21-22 | 0.19 | 0.014 | (0.04 to 0.34) |
|  | CpG23 | 0.07 | 0.38 | (-0.08 to 0.22) |
| Triceps | CpG1 | 0.07 | 0.42 | (-0.11 to 0.25) |
|  | CpG5-8 | 0.06 | 0.49 | (-0.11 to 0.23) |
|  | CpG11-12 | 0.17 | 0.08 | (-0.02 to 0.36) |
|  | CpG13-14 | 0.11 | 0.24 | (-0.07 to 0.29) |
|  | CpG21-22 | 0.21 | 0.012 | (0.05 to 0.38) |
|  | CpG23 | 0.13 | 0.12 | (-0.03 to 0.29) |
|  |  |  |  |  |
| **Anthropometry** |  |  |  |  |
| Weight | CpG1 | 0.04 | 0.62 | (-0.12 to 0.20) |
|  | CpG5-8 | -0.04 | 0.66 | (-0.193 to 0.122) |
|  | CpG11-12 | 0.065 | 0.468 | (-0.11 to 0.24) |
|  | CpG13-14 | 0.08 | 0.35 | (-0.09 to 0.24) |
|  | CpG21-22 | 0.11 | 0.15 | (-0.04 to 0.27) |
|  | CpG23 | 0.09 | 0.27 | (-0.07 to 0.24) |
| Height | CpG1 | 0.003 | 0.96 | (-0.13 to 0.14) |
|  | CpG5-8 | -0.10 | 0.14 | (-0.23 to 0.03) |
|  | CpG11-12 | -0.06 | 0.42 | (-0.20 to 0.08) |
|  | CpG13-14 | 0.06 | 0.36 | (-0.07 to 0.20) |
|  | CpG21-22 | -0.12 | 0.073 | (-0.24 to 0.01) |
|  | CpG23 | -0.06 | 0.38 | (-0.18 to 0.07) |
| BMI | CpG1 | 0.04 | 0.68 | (-0.13 to 0.20) |
|  | CpG5-8 | -0.002 | 0.98 | (-0.16 to 0.16) |
|  | CpG11-12 | 0.09 | 0.34 | (-0.09 to 0.26) |
|  | CpG13-14 | 0.05 | 0.57 | (-0.12 to 0.22) |
|  | CpG21-22 | 0.16 | 0.05 | (0.00 to 0.31) |
|  | CpG23 | 0.11 | 0.18 | (-0.05 to 0.26) |
| Waist | CpG1 | 0.13 | 0.15 | (-0.05 to 0.30) |
|  | CpG5-8 | -0.005 | 0.96 | (-0.17 to 0.16) |
|  | CpG11-12 | 0.16 | 0.08 | (-0.02 to 0.35) |
|  | CpG13-14 | 0.14 | 0.13 | (-0.04 to 0.31) |
|  | CpG21-22 | 0.10 | 0.21 | (-0.06 to 0.27) |
|  | CpG23 | 0.02 | 0.80 | (-0.14 to 0.18) |
|  |  |  |  |  |
| **Fat Thickness by Ultrasound measurement** |  |  |  |  |
| Subcutaneous | CpG1 | 0.14 | 0.087 | (-0.02 to 0.30) |
|  | CpG5-8 | 0.04 | 0.60 | (-0.11 to 0.19) |
|  | CpG11-12 | 0.16 | 0.058 | (-0.005 to 0.33) |
|  | CpG13-14 | 0.18 | 0.029 | (0.02 to 0.34) |
|  | CpG21-22 | 0.13 | 0.093 | (-0.02 to 0.28) |
|  | CpG23 | 0.08 | 0.26 | (-0.06 to 0.23) |
| Visceral Fat | CpG1 | -0.090 | 0.30 | (-0.26 to 0.08) |
|  | CpG5-8 | -0.12 | 0.15 | (-0.29 to 0.04) |
|  | CpG11-12 | -0.09 | 0.32 | (-0.27 to 0.09) |
|  | CpG13-14 | -0.12 | 0.17 | (-0.30 to 0.05) |
|  | CpG21-22 | 0.07 | 0.39 | (-0.09 to 0.23) |
|  | CpG23 | 0.05 | 0.50 | (-0.10 to 0.21) |
| Birth parameters |  |  |  |  |
| Birth weight | CpG1 | -0.06 | 0.39 | (-0.06 to 0.15) |
|  | CpG5-8 | 0.03 | 0.59 | (-0.08 to 0.14) |
|  | CpG11-12 | 0.02 | 0.82 | (-0.11 to 0.14) |
|  | CpG13-14 | -0.00 | 0.99 | (-0.12,0.12) |
|  | CpG21-22 | 0.02 | 0.68 | (-0.09 to 0.14) |
|  | CpG23 | 0.06 | 0.29 | (-0.05 to 0.17) |
| Birth length | CpG1 | -0.01 | 0.81 | (-0.11 to 0.09) |
|  | CpG5-8 | 0.00 | 0.92 | (-0.9 to 0.10) |
|  | CpG11-12 | -0.02 | 0.69 | (-0.14 to 0.09) |
|  | CpG13-14 | 0.00 | 0.92 | (-0.10 to 0.11) |
|  | CpG21-22 | -0.01 | 0.87 | (-0.11 to 0.09) |
|  | CpG23 | 0.02 | 0.75 | (-0.08 to 0.11) |
| Birth head circumference | CpG1 | -0.00 | 0.94 | (-0.12 to 0.11) |
|  | CpG5-8 | 0.06 | 0.31 | (-0.05 to 0.17) |
|  | CpG11-12 | -0.02 | 0.76 | (-0.15 to 0.11_ |
|  | CpG13-14 | -0.01 | 0.84 | (-0.12 to 0.10) |
|  | CpG21-22 | 0.03 | 0.61 | (-0.08 to 0.14) |
|  | CpG23 | 0.09 | 0.10 | -0.02 to 0.20 |

Supplementary Table 3: Multivariate models for birth anthropometry

| Birth **Anthropometry** | **CpG** | **β coefficient** | **P Value** | **(95% CI)** |
| --- | --- | --- | --- | --- |
| Birth Weight | CpG1 | -0.015 | 0.89 | -0.23 to 0.20 |
|  | CpG5-8 | 0.03 | 0.61 | -0.09 to 0.16 |
|  | CpG11-12 | 0.04 | 0.67 | -0.13 to 0.20 |
|  | CpG13-14 | 0.00 | 1.00 | -0.21 to 0.21 |
|  | CpG21-22 | -0.07 | 0.49 | -0.28 to 0.14 |
|  | CpG23 | 0.12 | 0.22 | -0.07 to 0.30 |
| Birth Length | CpG1 | -0.05 | 0.62 | -0.23 to 0.14 |
|  | CpG5-8 | 0.01 | 0.88 | -0.10 to 0.12 |
|  | CpG11-12 | -0.02 | 0.84 | -0.17 to 0.14 |
|  | CpG13-14 | 0.06 | 0.50 | -0.12 to 0.25 |
|  | CpG21-22 | -0.05 | 0.58 | -0.23 to 0.13 |
|  | CpG23 | 0.06 | 0.48 | -0.10 to 0.22 |
| Birth Head Circumference | CpG1 | -0.02 | 0.87 | -0.22 to 0.19 |
|  | CpG5-8 | 0.08 | 0.22 | -0.045 to 0.20 |
|  | CpG11-12 | -0.02 | 0.85 | -0.18 to 0.15 |
|  | CpG13-14 | -0.003 | 0.98 | -0.20 to 0.20 |
|  | CpG21-22 | -0.09 | 0.40 | -0.29 to 0.11 |
|  | CpG23 | 0.16 | 0.084 | -0.02 to 0.34 |

Supplementary Table 4: Eigenvectors for first two Principal Components of DNA Methylation.

| Variable | Component 1 | Component 2 |
| --- | --- | --- |
| CpG1 | 0.51 | -0.17 |
| CpG5-8 | 0.31 | -0.33 |
| CpG11-12 | 0.42 | -0.22 |
| CpG13-14 | 0.49 | -0.23 |
| CpG21-22 | 0.42 | 0.51 |
| CpG23 | 0.25 | 0.71 |
